# Altered miRNA expression in duodenal tissue of celiac patients and the impact of a gluten-free diet: a preliminary study

**DOI:** 10.1007/s11033-025-10534-y

**Published:** 2025-04-30

**Authors:** Zuzana Kolkova, Stanislava Suroviakova, Marian Grendar, Zuzana Havlicekova, Andrea Hornakova, Veronika Holubekova, Erika Halasova, Peter Banovcin

**Affiliations:** 1 Laboratory of Genomics and Prenatal Diagnostics, Biomedical Center Martin, Jessenius Faculty of Medicine in Martin, Comenius University in Bratislava, Martin, Slovakia; 2 Department of Pediatrics, Jessenius Faculty of Medicine in Martin, Comenius University in Bratislava, Martin, Slovakia; 3 Department of Pediatrics, University Hospital Martin, Martin, Slovakia; 4 Laboratory of Bioinformatics and Biostatistics, Biomedical Center in Martin, Jessenius Faculty of Medicine, Comenius University in Bratislava, Martin, Slovakia; 5 Biomedical Center Martin, Jessenius Faculty of Medicine in Martin, Comenius University in Bratislava, Martin, Slovakia

**Keywords:** MiRNA, Dysregulation, Celiac disease, Gluten-free diet, Molecular pathways

## Abstract

**Background:**

MicroRNAs (miRNAs) are crucial regulators of gene expression, impacting a wide range of biological processes. Their dysregulation can result in pathological changes and contribute to the development of various disorders. This study aims to evaluate the expression of selected miRNAs in duodenal tissue of paediatric patients with active celiac disease (CD), investigate the role of dysregulated miRNAs in disease pathogenesis and assess the changes in their expression profile in response to a gluten-free diet (GFD).

**Methods and results:**

The study included newly diagnosed celiac patients (*n* = 20), celiac patients adhering to a GFD (*n* = 17) and a control group (*n* = 29). The miRNA expression in duodenal samples was quantified by real-time PCR. Dysregulated miRNAs were analysed for functional enrichment in molecular pathways. Our results identified 8 dysregulated miRNAs in celiac patients: miR-155-5p (upregulated) and hsa-miR-22-5p, hsa-miR-192-5p, hsa-miR-338-3p, hsa-miR-31-5p, hsa-miR-31-3p, hsa-miR-215-5p and hsa-miR-378d (downregulated). Pathway analysis implicated these miRNAs in regulating various signaling pathways related to inflammation, immune response and intercellular junctions, all of which are relevant to the pathogenesis of CD. Moreover, miR-31-3p was upregulated in CD patients on a GFD, exhibiting a negative correlation with the duration of GFD. For other miRNAs, the level of expression in CD patients adhering to a GFD was restored to levels similar to those observed in the control group.

**Conclusion:**

This preliminary study reveals significant changes in miRNA expression in duodenal biopsies from paediatric CD patients and how these patterns shift with dietary intervention. Understanding the interactions among dysregulated miRNAs may lead to novel therapeutic strategies for managing CD.

**Supplementary Information:**

The online version contains supplementary material available at 10.1007/s11033-025-10534-y.

## Introduction

Celiac disease (CD) is a chronic autoimmune disorder that affects approximately 1% of the general population in Europe [1]. It is triggered by the ingestion of dietary gluten in genetically susceptible individuals and is characterized by an aberrant immune response, leading to intestinal inflammation, villous atrophy, crypt hyperplasia and lymphocyte infiltration [2]. Although the HLA DQ2 and DQ8 haplotypes are the main genetic factors necessary for CD development, they are not sufficient, as these haplotypes are present in 30–40% of the general population, but only 1% of haplotype carriers develop CD [3]. Based on GWAS (genome-wide association studies), other HLA-related and non-HLA loci implicated in immune system function have been identified as risk factors for CD development [4, 5]. The genetic background of this disease is complex, and although genetics plays a key role in the pathogenesis of CD, it is not the only contributing factor. The pathogenesis of CD involves complex interactions between genetic predisposition, environmental factors and immune responses. While the genetic factors are well characterised, the role of epigenetics, especially microRNAs (miRNAs), in this complex disorder has received increased attention in recent years. McKenna et al. demonstrated the essential role of miRNAs in maintaining intestinal homeostasis. In their work, a deficiency of Dicer, a crucial protein in miRNA biogenesis, negatively influenced intestinal epithelial organization, reducing the number of Goblet cells and increasing apoptosis in crypts in a mouse model. Additionally, a lack of Dicer led to inflammation, with lymphocyte and neutrophil infiltration as a consequence of epithelial barrier disruption [6].

MicroRNAs (miRNAs) are a class of small, non-coding RNAs that play crucial roles in post-transcriptional gene regulation by binding to the 3’-untranslated regions (UTRs) of target mRNAs, resulting in either mRNA degradation or translational repression. MiRNAs have been implicated in various biological processes, including development, differentiation, proliferation, apoptosis and immune responses [6, 7]. Dysregulation of miRNA expression has been linked to several autoimmune diseases, underscoring their potential as diagnostic markers and therapeutic targets, as they may provide insight into disease activity and response to treatment [8]. In the context of CD, research on miRNA expression profiles has helped to elucidate their involvement in the complex interplay of immune responses, inflammation and tissue damage. Several studies have investigated the expression patterns of miRNAs in CD patients, in both circulating blood and intestinal biopsies, and have identified dysregulated miRNAs that may contribute to the development and progression of the disease [9–15]. MiRNAs are known to influence the expression of key genes involved in CD pathogenesis, such as those related to innate and adaptive immunity, intestinal barrier integrity, and gluten metabolism [16–18], so they have been proposed as potential diagnostic and prognostic markers for CD. However, despite the growing body of evidence suggesting the significance of miRNAs in CD, a comprehensive understanding of their precise roles and mechanisms of action remains unclear.

In this manuscript, we present our pilot study aimed at comparing miRNA expression levels in duodenal tissue among three groups: active celiac patients, CD patients adhering to a gluten-free diet (GFD) and non-celiac controls. Our objective was to assess the role of these miRNAs in the pathogenesis of CD. A panel of tested miRNAs was designed based on previous studies, focusing on their potential involvement in autoimmune diseases, immune regulation, inflammation, epithelial barrier function, tissue repair and the response to a GFD [9–13, 19–31]. We hope to contribute to a deeper understanding of the molecular basis of this complex disorder and identify new possibilities for future research and therapeutic interventions.

## Materials and methods

### Study groups and sample collection

A total of 66 participants of Caucasian ethnicity, 40 girls and 26 boys, aged 2–19 years were included in the study approved by the Ethical committee (EK 60/2018) of Jessenius Faculty of Medicine in Martin, Comenius University in Bratislava. The patients and their parents were informed about the study and signed an informed consent in agreement with the Declaration of Helsinki. Twenty patients were newly diagnosed with CD, and 29 participants with excluded CD were included in the control group. A third group, including 17 celiac patients who had been on a GFD for more than 2 years, was created to monitor the miRNA expression of selected miRNAs as a response to the absence of gluten in the diet. All the participants underwent a screening test for tissue transglutaminase antibodies (tTGA) and esophagogastroduodenoscopy with duodenal biopsy. At least 4 biopsies from the distal duodenum and at least 1 from the duodenal bulb were taken for histology assessment using endoscopic forceps. Histological lesions in CD were graded using the classification system of Corazza and Villanacci [32]. Diagnosis of CD was confirmed according to the European Society for Paediatric Gastroenterology, Hepatology and Nutrition Guidelines for Diagnosing Coeliac Disease 2020 [33]. Participants included in the control group underwent esophagogastroduodenoscopy for other reasons (e.g. foreign body ingestion). Diagnosis of CD in control group was ruled out on the basis of negative histological findings and tissue transglutaminase antibodies. The inclusion criteria for CD patients in the GFD group were, besides more than 2 years on a gluten-free diet, negative autoantibodies for tTGA and histologically confirmed grade Marsh 0. The exclusion criteria for this study included the presence of food intolerances, other gastrointestinal disorders, such as *Helicobacter pylori* infection or other inflammatory conditions, as well as using probiotics, immunosuppressants or corticosteroids at the time of biopsy sampling.

### RNA isolation and quantification

Total RNA, including the miRNA fraction, was isolated from frozen (−80 °C) duodenal biopsies stored in RNAprotect Tissue Reagent (Qiagen, Germany) using the miRVana miRNA Isolation Kit according to the manufacturer’s instructions (Invitrogen, USA). Extracted RNA was quantified with the Qubit RNA BR kit (Thermo Fisher Scientific) on a Qubit 4 Fluorometer (Thermo Fisher Scientific).

### MiRNA expression measurement by qPCR

The expression levels of selected miRNAs, as detailed in Table 1, were quantified using the RT-qPCR method with the miRCURY LNA miRNA PCR System (Qiagen, Germany). Technical variability between samples was controlled by using spike-in UniSp6 added to the reverse transcription reaction. All candidate miRNAs, along with the potential reference miRNAs (SNORD48 and RNU6), were quantified in duplicate using specific miRCURY LNA miRNA assays (Qiagen, Germany) (Table 1) on the LightCycler 480 Instrument (Roche, Germany). The Cq (quantification cycle) values were determined by the second derivative method using the LightCycler480 Instrument software [34]. The technical variability of the reverse transcription reaction was assessed based on the UniSp6 spike-in expression level using the GeneGlobe analysis tool (https://geneglobe.qiagen.com/us/). Additionally, the GeneGlobe was utilized to calculate the stability factors of reference genes through geNorm analysis [35]. The expression levels were calculated using the 2^-∆∆Ct^ method.

**Table 1:** List of studied MiRNAs

miRNA	Assay catalog number	Mature miRNA sequence
hsa-miR-155-5p	YP00204308	5’UUAAUGCUAAUCGUGAUAGGGGU
hsa-miR-103a-3p	YP00204063	5’AGCAGCAUUGUACAGGGCUAUGA
hsa-miR-151b	YP00204007	5’UCGAGGAGCUCACAGUCU
hsa-miR-378d	YP02100497	5’ACUGGACUUGGAGUCAGAAA
hsa-miR-30a-5p	YP00205695	5’UGUAAACAUCCUCGACUGGAAG
hsa-miR-338-3p	YP00204719	5’UCCAGCAUCAGUGAUUUUGUUG
hsa-miR-215-5p	YP00204598	5’AUGACCUAUGAAUUGACAGAC
hsa-miR-200c-3p	YP00204482	5’UAAUACUGCCGGGUAAUGAUGGA
hsa-miR-107	YP00204468	5’AGCAGCAUUGUACAGGGCUAUCA
hsa-miR-652-3p	YP00204387	5’AAUGGCGCCACUAGGGUUGUG
hsa-miR-28-5p	YP00204322	5’AAGGAGCUCACAGUCUAUUGAG
hsa-miR-22-5p	YP00204255	5’AGUUCUUCAGUGGCAAGCUUUA
hsa-miR-31-5p	YP00204236	5’AGGCAAGAUGCUGGCAUAGCU
hsa-miR-26b-5p	YP00204172	5’UUCAAGUAAUUCAGGAUAGGU
hsa-miR-192-5p	YP00204099	5’CUGACCUAUGAAUUGACAGCC
hsa-miR-151a-5p	YP00204007	5’UCGAGGAGCUCACAGUCUAGU
hsa-miR-31-3p	YP00204079	5’UGCUAUGCCAACAUAUUGCCAU
U6 snRNA	YP00203907	
SNORD48	YP00203903	
UniSp6	YP00203954	

### Functional enrichment analysis of dysregulated miRNAs

Functional analysis of dysregulated miRNAs was conducted using miRNA-centric analysis of the web-based tool miRPath v4.0 (available at http://www.microrna.gr/miRPathv4) [36]. Pathway enrichment analysis was performed using the Kyoto Encyclopedia of Genes and Genomes (KEGG) with the gene union method for 8 miRNAs with a below FC threshold cut-off of 0.7, namely hsa-miR-155-5p, hsa-miR-31-5p, hsa-miR-31-3p, hsa-miR-22-5p, hsa-miR-338-3p, hsa-miR-192-5p, hsa-miR-215-5p and hsa-miR-378d. TarBase v8.0 was selected as a source of experimentally verified miRNA-target interactions. The enrichment analysis was performed using the hypergeometric test to evaluate the significance of the pathway association. P values were adjusted for the false discovery rate (FDR), and pathways with an FDR < 0.05 were considered statistically significant, indicating a strong relationship between specific miRNAs and target genes in these pathways.

### Bioinformatic analysis and statistics

We used R version 4.0.5 [37] and several R packages, with robustbase v.0.99.2 [38] and ggpubr v. 0.4.0.999 [39] being the most prominent ones. Changes in miRNA expression were expressed as a log_2_ fold change (log_2_FC) between two conditions calculated by the standard formula [40] and visualized by boxplots. To analyse the relationship between two quantitative variables (miRNA expression and duration of GFD), we used Spearman’s correlation analysis after testing data normality by the Shapiro-Wilk test in the software SYSTAT 13 (Systat Software Inc., USA). Results with *p* < 0.05 were considered statistically significant.

## Results

### Characteristics of the study cohort

The study cohort (*n* = 66) consisted of three groups: 20 patients (6 males and 14 females) with newly diagnosed CD aged 2–15 years, 29 healthy controls (12 males and 17 females) ranging in age from 2 to 18 years, and 17 GFD patients (7 males and 10 females), age range 15–19 years with duration of GFD more than 2 years (range 2–16 years). Based on the histological classification of Corazza and Villanacci [32], 50% of CD patients were classified as Grade B1 and 50% as Grade B2. The basic demographic and clinical characteristics are listed in Table 2.

**Table 2:** The basic characteristics of the study cohort. The median, with the corresponding minimum and maximum in brackets or a number and percent in brackets, is present for each variable

	CD patients (*n* = 20)	Controls (*n* = 29)	GFD (*n* = 17)
Age (years)	9.5 (2–15)	10 (2–18)	19 (15–19)
Gender			
Male	6 (30%)	12 (41.4%)	7 (41.2%)
Female	14 (70%)	17 (58.6%)	10 (58.8%)
Age at diagnosis (years) Duration of GFD (years)	9.5 (2–15) -	- -	12 (2–17) 6 (2–16)
Histological classification			
Grade B1	10 (50%)	-	0
Grade B2	10 (50%)	-	0

CD - celiac disease, GFD - gluten-free diet

### MiRNA expression profiles of Celiac disease patients

**Fig. 1:**
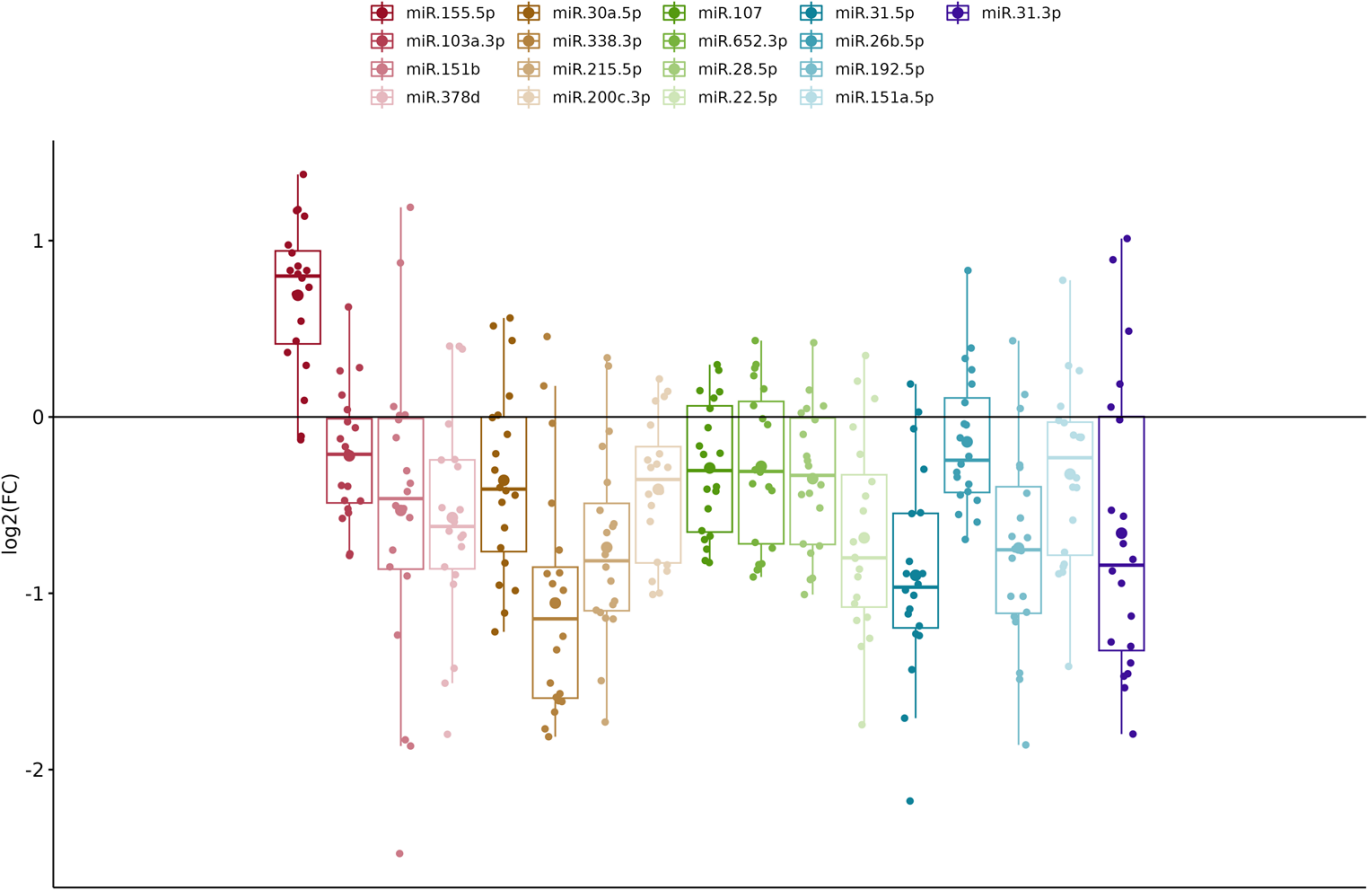
Boxplots illustrating the log_2_ fold change (log_2_FC) in miRNA expression levels in the CD group compared to the control group. Each boxplot represents the distribution of log_2_FC values for specific miRNAs

The acquired Cq values were normalized to two housekeeping genes, namely U6 and SNORD48, ensuring reliable quantification and minimizing variability across samples. The combination of these two miRNAs was evaluated as the most stable across the study groups by geNorm analysis [35], provided in the GeneGlobe tool. The average arithmetic means for the celiac patient group was 30.74, for the control group 30.6 and for the GFD group 30.67. A statistical analysis of the expression levels of 17 measured miRNAs in patients with active CD, expressed as a FC compared to healthy controls, revealed statistically significant differences in 16 miRNAs. In particular, no significant difference was observed for hsa-miR-26b-5p. Among the miRNAs, hsa-miR-155-5p exhibited upregulation with a FC of 1.74, while the remaining 15 miRNAs were downregulated. Detailed results are presented in Table 3. The expression data are visualized by boxplots based on log_2_FC to provide a symmetrical interpretation of the results (Fig. 1). Although statistical significance was achieved, the differences in expression levels were often very slight. To assess the biological function of dysregulated miRNAs in the process of CD development, a fold-change threshold cut-off of 0.7 was established to select downregulated miRNAs for further pathway enrichment analysis. This threshold indicates that miRNAs with a FC below this cut-off exhibited a more than 30% reduction in expression compared to the control group. The selected miRNAs meeting this criterion along with one upregulated miRNA are highlighted in bold in the table. This approach allows for a focused analysis on miRNAs that may play significant roles in modulating the mechanisms implicated in disease pathogenesis.

**Table 3:** Differences in the expression of miRNAs in active celiac patients compared to non-celiac controls expressed as fold change (FC)

	Celiac disease patients (*n* = 20)		
	FC	log_2_ FC	*p* value
**hsa-miR-155-5p**	**1.74**	0.8	**0.00002**
hsa-miR-103a-3p	0.86	-0.21	**0.02148**
hsa-miR-151b	0.73	-0.46	**0.00944**
**hsa-miR-378d**	**0.65**	-0.62	**0.00048**
hsa-miR-30a-5p	0.75	-0.41	**0.01362**
**hsa-miR-338-3p**	**0.45**	-1.14	**0.00002**
**hsa-miR-215-5p**	**0.57**	-0.82	**0.00004**
hsa-miR-200c-3p	0.78	-0.35	**0.00026**
hsa-miR-107	0.81	-0.3	**0.00186**
hsa-miR-652-3p	0.81	-0.31	**0.01208**
hsa-miR-28-5p	0.8	-0.33	**0.00199**
**hsa-miR-22-5p**	**0.58**	-0.8	**0.00008**
**hsa-miR-31-5p**	**0.51**	-0.97	**0.00001**
hsa-miR-26b-5p	0.84	-0.25	0.08969
**hsa-miR-192-5p**	**0.59**	-0.75	**0.00005**
hsa-miR-151a-5p	0.85	-0.23	**0.00946**
**hsa-miR-31-3p**	**0.56**	-0.84	**0.00422**

FC- fold change value is expressed as median, p value < 0.05 is considered as statistically significant

A robust regression analysis was employed to assess the potential impact of other factors, specifically age, gender and histological grade, on the expression level of the analysed miRNAs in celiac patients. This model revealed no statistically significant association of basic demographic and clinical parameters with the expression of any measured miRNAs, which increased the validity of our results. P values are listed in the Supplementary Material 1.

### Pathway enrichment analysis

**Fig. 2:**
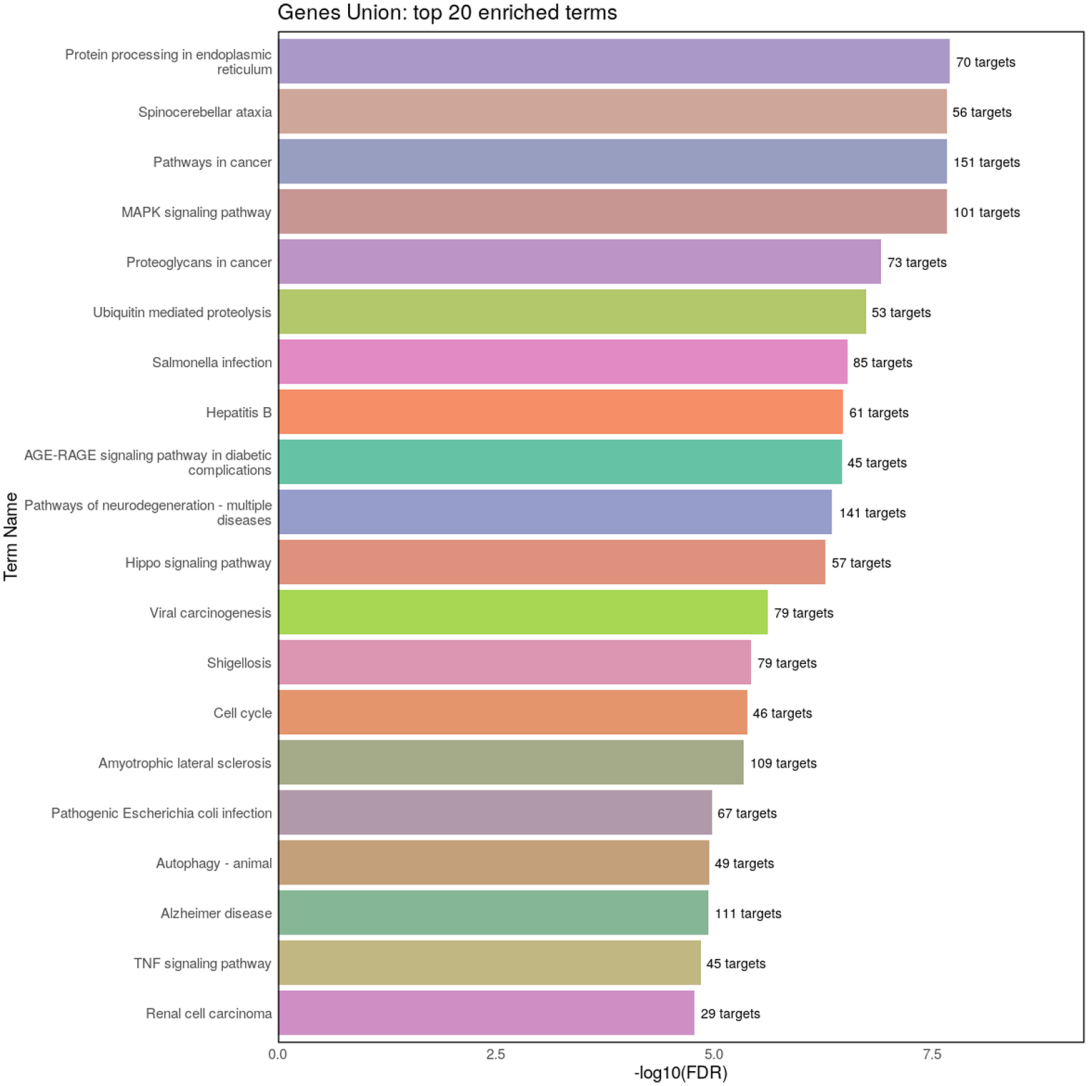
The 20 most enriched KEGG pathways from gene union analysis provided by miRPath v4.0

The role of selected dysregulated miRNAs, namely hsa-miR-155-5p, hsa-miR-22-5p, hsa-miR-192-5p, hsa-miR-338-3p, hsa-miR-31-5p, hsa-miR-31-3p, hsa-miR-215-5p and hsa-miR-378d, in the regulation of various biological pathways was discovered through KEGG pathway analysis. The analysis revealed 122 KEGG pathways with an FDR < 0.05. Twenty of the most enriched pathways with the number of corresponding miRNA-targeted genes are displayed in Fig. 2, and the complete list is attached in the Supplementary Material 2. Among others, targeted genes were enriched in pathways related to signal transduction (e.g. MAPK, Hippo, TNF, p53, PI3K-Akt, Wnt, TGF-beta, JAK-STAT and the Hedgehog signaling pathway), immune system (T-cell receptor signaling pathway, IL-17 signaling pathway, NOD-like receptor signaling pathway), cellular community (adherens junction, focal adhesion, tight junction, gap junction), apoptosis or bacterial invasion of epithelial cells, which are relevant for CD pathogenesis.

### Effect of a gluten-free diet on the miRNA expression profile

**Fig. 3:**
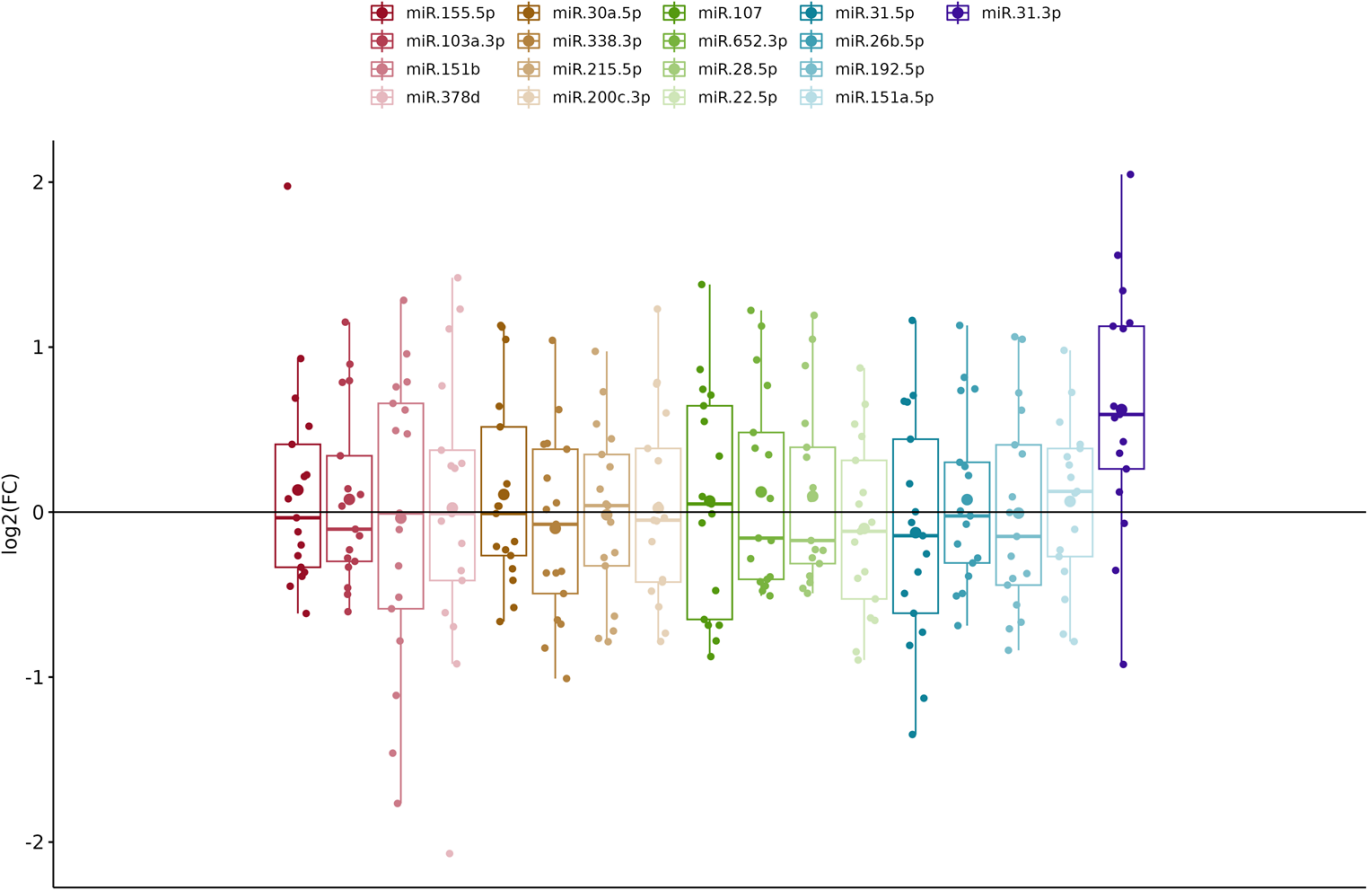
Boxplots presenting the log_2_ fold change (log_2_FC) in miRNA expression levels between celiac patients on a gluten-free diet and the control group. Each boxplot illustrates the distribution of log_2_FC values for specific miRNAs

Duodenal tissue samples of 17 celiac patients following a GFD for more than 2 years were included in the GFD group to investigate the effect of a GFD on the expression levels of the studied miRNAs. Comparing their expression level to those of non-celiac controls, many miRNAs exhibited no significant changes with FC close to 1, indicating minimal variation in expression. Only hsa-miR-31-3p showed statistically significant upregulation with FC 1.51 (pval 0.00258) in the GFD group compared to controls. The overall results are summarised in Table 4. and illustrated by boxplots based on log_2_FC (Fig. 3).

**Table 4:** MiRNA expression levels in celiac patients on GFD expressed as FC compared to non-celiac controls

	Celiac patients on GFD (*n* = 17)		
	FC	log_2_FC	*p* value
hsa-miR-155-5p	0.98	-0.03	0.7119
hsa-miR-103a-3p	0.93	-0.1	0.88997
hsa-miR-151b	0.99	-0.01	1.0
hsa-miR-378d	0.99	-0.01	0.88997
hsa-miR-30a-5p	0.99	-0.01	0.8498
hsa-miR-338-3p	0.95	-0.07	0.54767
hsa-miR-215-5p	1.03	0.04	0.92651
hsa-miR-200c-3p	0.97	-0.05	0.92651
hsa-miR-107	1.03	0.05	0.78191
hsa-miR-652-3p	0.9	-0.16	0.6777
hsa-miR-28-5p	0.89	-0.17	0.78191
hsa-miR-22-5p	0.92	-0.12	0.45857
hsa-miR-31-5p	0.91	-0.14	0.48738
hsa-miR-26b-5p	0.98	-0.02	0.81758
hsa-miR-192-5p	0.9	-0.15	0.88997
hsa-miR-151a-5p	1.09	0.13	0.57906
**hsa-miR-31-3p**	**1.51**	0.59	**0.00258**

FC- fold change value is expressed as median, p value < 0.05 is considered statistically significant

**Fig. 4:**
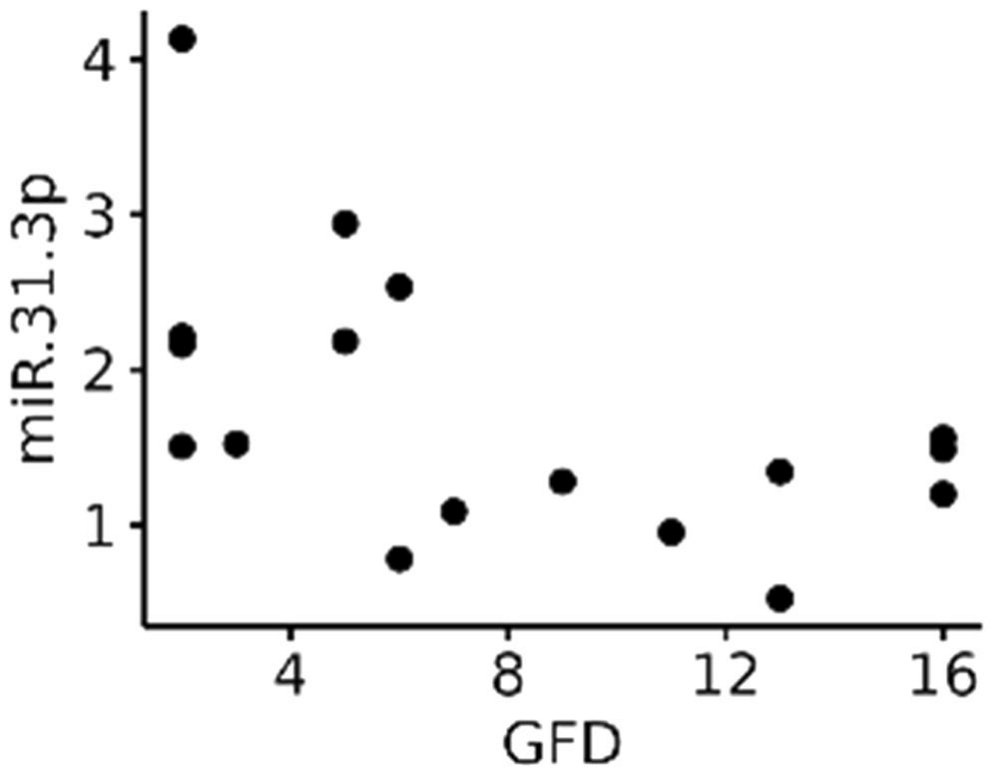
Scatterplot illustrating the relationship between miR-31-3p expression and the duration of GFD in celiac patients. Each point on the plot represents an individual patient with the duration of GFD adherence (in years) on the x axis and the miRNA-31-3p expression level as a FC relative to non-celiac controls

The relationship between the miRNA expression level and the duration of GFD adherence in celiac patients was investigated through correlation analysis. The Spearman correlation coefficients (R) revealed negative correlations for all analysed miRNAs, but only in the case of hsa-miR-31-3p, the correlation reached statistical significance (*R* = −0.57, *p* = 0.017). The results are summarized in Table 5. As shown in the scatterplot (Fig. 4), the expression of hsa-miR-31-3p in patients with a shorter duration of GFD adherence tended to be higher. We speculate on its function in ongoing epithelial renewal processes below.

**Table 5:** Correlation of miRNA expression with the duration of GFD expressed by Spearman correlation coefficient (R). Statistically significant results (*p* < 0.05) are highlighted in bold

	*R*	*p* value
hsa-miR-155-5p	-0.146	0.576
hsa-miR-103a-3p	-0.332	0.193
hsa-miR-151b	-0.427	0.087
hsa-miR-378d	-0.291	0.257
hsa-miR-30a-5p	-0.379	0.134
hsa-miR-338-3p	-0.277	0.282
hsa-miR-215-5p	-0.348	0.171
hsa-miR-200c-3p	-0.301	0.24
hsa-miR-107	-0.149	0.568
hsa-miR-652-3p	-0.379	0.134
hsa-miR-28-5p	-0.289	0.261
hsa-miR-22-5p	-0.426	0.088
hsa-miR-31-5p	-0.365	0.15
hsa-miR-26b-5p	-0.259	0.315
hsa-miR-192-5p	-0.329	0.197
hsa-miR-151a-5p	-0.317	0.215
**hsa-miR-31-3p**	**-0.57**	**0.017**

## Discussion

The pathogenesis of CD is complex and involves many genetic, environmental and immune factors. Through a collaborative effort, research teams aim to uncover and understand the pathomechanisms of the disease and explore new opportunities for its diagnosis, management and treatment. Celiac disease can manifest itself at any age, although the initial disease trigger is still not clear. One of the modulating factors is the regulation of gene expression by miRNAs. Previous studies have demonstrated the involvement of miRNAs in immune response, inflammation, epithelial barrier integrity and intestinal homeostasis. Dysregulation of miRNAs has been studied in the context of CD, its severity, clinical manifestation, age of manifestation and adherence to a GFD both in tissue samples and the blood circulation, in paediatric and adult patients [9–13, 15, 41–43].

In our pilot study, we detected 1 upregulated and 15 downregulated miRNAs in duodenal biopsies of celiac patients. Furthemore, we focused on the 8 most differently expressed miRNAs (upregulated: hsa-miR-155-5p; downregulated: hsa-miR-22-5p, hsa-miR-192-5p, hsa-miR-338-3p, hsa-miR-31-5p, hsa-miR-31-3p, hsa-miR-215-5p and hsa-miR-378d) that may play significant roles in modulating the mechanisms implicated in CD pathogenesis. We included these miRNAs in pathway analysis, and discuss them below. We also analysed the correlation of all miRNAs with factors such as age, gender and the severity of intestinal damage in a regression model. Although previous studies reported that the expression of specific miRNAs may vary based on age [12], disease manifestation [13] or grade [11], our regression model revealed no statistically significant associations between these basic parameters and the expression levels of the tested miRNAs.

Overall, one of the best studied and characterized miRNAs is miR-155, which is linked to the immune system, inflammation and autoimmune diseases, as well as cancer. It is highly expressed in the various immune cells, including macrophages, T cells and B cells, where it regulates differentiation and function [19, 44], promotes differentiation of T-helper cells [19], especially those of the Th-1 and Th-17 lineages, which are implicated in inflammatory processes in CD pathogenesis [45]. Moreover, overexpression of miR-155-5p was associated with increased intestinal permeability in an experimental model of acute pancreatitis [46], dextran sulpfate sodium (DSS)-induced colitis [47] and irritable bowel syndrome [48]. Bascuñán et al. reported elevated levels of miR-155 in peripheral blood of adult patients with active CD as well as in CD patients on a GFD for at least 1 year. Their results suggest that increased miR-155 expression is not related to gluten intake [15]. A combined approach based on sequencing mRNA and miRNA in duodenal biopsies of paediatric CD patients identified upregulated miR-155-5p associated with regulation of mRNA transcripts related to immune response [9]. We have observed 1.74-fold upregulation of miR-155-5p in duodenal tissue samples of celiac patients compared to nonceliac controls, which emphasizes its proinflammatory potential and role in the regulation of immune response. Moreover, we observed normalization of miR-155-5p level in duodenal tissue after more than 2 years of GFD adherence.

Little is known about miR-378d and miR-22-5p, both downregulated in our group of CD patients. MiR-378d is a member of miR-378 family associated with apoptosis, cell proliferation, cell migration and carcinogenesis [49–52]. Studies on miR-378d function are limited. Zhu et al. described the relationship between *Mycobacterium tuberculosis* infection and miR-378d expression. They found that decreased expression of miR-378d in macrophages during infection led to upregulation of Rab10 through activation of the NF-κB signaling pathway and induction of proinflammatory cytokines. These results suggest the role of miR-378d in the modulation of immune response [21]. There are no specific studies about the role of miR-22-5p in the pathogenesis of CD. Future research focusing on the expression levels of the miR-22-5p in celiac patients, as well as the identification its target genes and pathways, will be essential to clarify their role in disease pathogenesis. Tan et al. in their comprehensive study concerning to miRNA- transcript networks in CD patients, identified that downregulated miRNA-22-5p targets transcripts involved in lymphocyte differentiation and IFN-γ signaling that are dysregulated in CD [9].

Our results showed significant downregulation of miR-192-5p, miR-31-5p, miR-338-3p and miR-215-5p in bioptic samples of paediatric CD patients. Monitoring the effect of GFD, we observed comparable expression levels of these miRNAs in treated CD patients and the nonceliac group, suggesting that elimination of gluten from the diet restores miRNA expression. Comparable results had been reported previously. Buoli Comani et al. [12] found underexpression of miR-31-5p and miR-338-3p in duodenal biopsies of paediatric CD patients, and a lower expression of plasma miRNA-192-5p and miR-31-5p compared to the control group, consistent with the severity of mucosal damage. In addition, the expression level of circulating miRNA-192-5p was persistently dysregulated in CD patients on GFD, whereas plasma miR-31-5p tented to return to normal levels after at least 1 year on a GFD. Decreased expression of miR-192-5p, miR-31-5p and miR-338-3p was also observed in duodenal samples of adult CD patients, which can lead to increased expression of targets involved in innate and adaptive immunity [11]. On the other hand, Felli et al. demonstrated upregulation of circulating miR-215-5p and miR-192-5p in the CD patient group compared to the control group likewise to the patient group on a GFD [10].

In contrast to miR-31-5p, miR-31-3p has not been well characterised. In the context of CD, its downregulation and implication in lymphocyte differentiation and interferon signaling were reported [9]. In our study, we detected a significant downregulation of miR-31-3p in duodenal tissues of CD patients. Among patients adhering to a GFD, we found a negative correlation between the duration of diet adherence and the miR-31-3p expression level. Notably, in the initial years following diet introduction, the expression level of this miRNA increased, possibly associated with changes in immune pathways as well as healing of the intestinal mucosa. The role of miR-31 in intestinal homeostasis and regeneration was previously described in a mouse model. Tian et al. reported high expression of miR-31 in the crypts, where it activates the canonical Wnt signaling pathway to promote proliferation of stem cells and to prevent their apoptosis in order to maintain intestinal homeostasis. Moreover, activation of the canonical Wnt pathway by miR-31 in the small intestine is a critical mechanism of epithelial regeneration [28]. Fang et al. detected significant overexpression of miR-31-3p in biopsies of ulcerative colitis patients compared to controls, but not in the case of Crohn’s disease biopsies. In an experimental mouse model of chemically induced colitis, miR-31-3p silencing led to worsening of colitis, mucosal damage and increased expression of cytokines. Based on their experiments on human colonocytes, miR-31-3p is involved in the regulation of cytokine expression by targeting RhoA and in maintaining mucosal homeostasis under inflammatory conditions [25]. Our pathway analysis has underscored the potential role of miR-31-3p in maintaining intercellular junctions and epithelial integrity. Based on this, we hypothesized that the increased expression of miR-31-3p may also be linked to the restoration of epithelial barrier function. Previous studies have indicated that long-term adherence to GFD only partially restores impaired intestinal barrier function in celiac patients [53, 54]. In future studies, it would be valuable to investigate the gene expression of miR-31-3p target genes involved in the Wnt pathway and those related to the maintenance of epithelial barrier integrity in duodenal tissue of CD patients. This could provide crucial insights into the role of miR-31-3p in intestinal homeostasis and promoting mucosal healing in CD.

Our study provides valuable insight into their contribution to the complex interplay of signaling pathways involved in CD development. Many pathways were related to the immune system, inflammation, intercellular junctions and signaling pathways associated with homeostasis, proliferation and development of the intestinal epithelium. For example, pathways related to T-cell receptor signaling and cytokine production may help explain how dysregulated miRNAs contribute to abnormal immune reactions observed in celiac patients. Furthermore, pathways involved in the function of the epithelial barrier highlight potential mechanisms through which modification of miRNA expression can lead to increased intestinal permeability and subsequent immune activation. The results of pathway analysis showed that all 8 analysed miRNAs target genes that are implicated in epithelial barrier integrity, which is critical for maintaining immune homeostasis and preventing inappropriate immune responses to gluten. In patients with CD, several structural and functional changes as a result of dysregulated gene expression of tight junction proteins [55–57], as well as in adherens junction proteins [56], have been observed. Although gluten is considered the primary factor inducing the changes in integrity and function of epithelial barrier, other factors are also implicated in this process. Further research into miRNAs, their specific interactions and the mechanisms regulating paracellular permeability could provide insight into CD pathogenesis and the restoration of epithelial barrier function.

This was a pilot study focused on elucidating the changes in the expression levels of various miRNAs in CD patients and patients adhering to a GFD, as well as exploring their enrichment in molecular pathways. The primary aim of this study was to investigate miRNA profiles specific to the paediatric population, as this group presents unique diagnostic and therapeutic challenges. Paediatric patients often experience more severe intestinal damage, such as Marsh 3 C lesions, along with acute and pronounced gastrointestinal symptoms. Unlike adults, children are typically diagnosed during the active phase of the disease, which may significantly influence miRNA expression patterns. Previous studies have demonstrated that the expression levels of certain genes and miRNAs differ between paediatric and adult CD patients [12, 58]. For example, miRNAs such as miR-192-5p and miR-31-5p are dysregulated in children with CD, reflecting distinct molecular mechanisms linked to immune responses and tissue repair [12]. These differences may be attributed to age-related factors, including variations in gut architecture, microbiota composition, and immune system maturation, all of which can affect intestinal and immune responses to gluten exposure [59]. Our findings are specific to paediatric patients, and future studies comparing these results with adult populations will be necessary to determine whether the identified miRNAs are applicable across age groups. The strengths of our study are in the identification of significant changes in miRNA expression in the active stage of CD and highlighting their involvement in molecular pathways regulating inflammation, immune response and epithelial barrier function contributing to disease pathogenesis. In the future, it would be beneficial to conduct longitudinal studies monitoring miRNA expression in relation to various clinical parameters, symptoms, and patient outcomes over time to determine whether specific miRNAs can serve as biomarkers of disease severity or predictors of long-term outcomes, such as mucosal healing and adherence to GFD. Moreover, we have also assessed the impact of adherence to a GFD on the modulation of miRNA expression levels during treatment as studies monitoring tissue miRNA expression as a response to GFD in paediatric CD patients are missing. On the other hand, the limitation of our study is the limited number of patients in the study groups. The small sample size in our study highlights the difficulties of obtaining duodenal biopsies in paediatric patients. The current guidelines of ESPGHAN [33] highly recommend to minimize invasive procedures, such as endoscopic duodenal biopsis, especially in cases when serological test strongly suggest CD. This approach toward non-invasive methods has resulted in fewer biopsies being conducted in clinical practice, which in turn limits the availability of biopsy samples for research. It is essential to validate our results in an independent and larger cohort to increase the value and reproducibility of our findings. Another limitation of our study is the absence of gene expression analysis of target genes identified by pathway analysis. Future research should aim to elucidate the functional roles of these miRNAs and their interactions with target genes to better understand their contributions to CD pathogenesis. In this study, we analysed miRNA expression in samples of whole duodenal tissue, which limits the ability to assess potential differences in expression levels in various cell types, such as immune and epithelial cells. Since miRNA expression may vary between cell types based on their specific function, the overall miRNA profile may be distorted. In a follow-up study, it would be beneficial to evaluate the expression of these miRNAs together with the expression of their target genes in individual cell types to assess their function and contribution to disease mechanisms.

## Conclusion

This preliminary study provides a valuable insight into changes in miRNA expression observed in duodenal biopsies of paediatric patients with active CD, as well as the alterations in their expression pattern in response to adherence to a GFD. Our findings suggest the potential role of these miRNAs in the pathogenesis of the disease, as the pathway analysis indicates their involvement in critical processes such as immune response, signaling pathways involved in inflammation and intestinal homeostasis or maintaining epithelial barrier integrity. Our findings contribute to the understanding of CD pathogenesis and could aid future advanced research and clinical and pharmaceutical interventions. In addition, a deeper understanding of the specific interactions of dysregulated miRNAs could open the way for novel therapeutic strategies and interventions in CD management.

## Electronic supplementary material

Below is the link to the electronic supplementary material.

Supplementary Material 1

Supplementary Material 2

## Data Availability

No datasets were generated or analysed during the current study.

## Abbreviations

CD: Celiac disease
Cq: Quantification cycle
DSS: Dextran sulfate sodium
FC: Fold change
FDR: False discovery rate
GFD: Gluten-free diet
GWAS: Genome-wide association studies
HLA: Human leukocyte antigen
IFNγ: Interferon gamma
IL: Interleukin
JAK-STAT: Janus kinase/signal transducers and activators of transcription
KEGG: Kyoto Encyclopedia of Genes and Genomes
MAPK: Mitogen-activated protein kinase
miRNA: Microrna
mRNA: Messenger ribonucleic acid
NF-κB: Nuclear factor κB
NOD: Nucleotide-binding oligomerization domain
PI3K-Akt: Phosphatidylinositol 3‑kinase (PI3K)/protein kinase B (AKT)
RNA: Ribonucleic acid
RT-qPCR: Quantitative real-time polymerase chain reaction
TGF: Transforming growth factors
Th: T helper
TNF: Tumor necrosis factor
tTGA: Tissue transglutaminase antibody
UTR: Untranslated region
