# Supplementary material for: Altered miRNA expression in duodenal tissue of celiac patients and the impact of a gluten-free diet: a preliminary study
Source: Mol Biol Rep. 2025 Apr 30;52(1):441. doi: 10.1007/s11033-025-10534-y (PMC12043776; doi:10.1007/s11033-025-10534-y)
Supplement: Supplementary file 1 — Supplementary Material 1 [file 11033_2025_10534_MOESM1_ESM.docx]

**Altered miRNA expression in duodenal tissue of celiac patients and the impact of a gluten-free diet: a preliminary study**

Journal name: Molecular Biology Reports, Collection: Digestive diseases

Zuzana Kolkova, Stanislava Suroviakova, Marian Grendar, Zuzana Havlicekova, Andrea Hornakova, Veronika Holubekova, Erika Halasova, Peter Banovcin

*Corresponding author: Stanislava Suroviakova, Department of Pediatrics, Jessenius Faculty of Medicine in Martin, Comenius University in Bratislava, Martin, Slovakia, e mail:* *[stanislava.suroviakova@gmail.com](mailto:stanislava.suroviakova@gmail.com)*

***Supp Tab 1*** *Robust regression model incorporating age, gender, histological grade and fold change of miRNA expression in celiac patients related to the control group.*

|  | Age | Gender | Grade |
| --- | --- | --- | --- |
| hsa-miR-155-5p | 0.139 | 0.572 | 0.487 |
| hsa-miR-103a-3p | 0.775 | 0.945 | 0.816 |
| hsa-miR-151b | 0.693 | 0.953 | 0.307 |
| hsa-miR-378d | 0.81 | 0.408 | 0.927 |
| hsa-miR-30a-5p | 0.648 | 0.549 | 0.136 |
| hsa-miR-338-3p | 0.389 | 0.972 | 0.652 |
| hsa-miR-215-5p | 0.893 | 0.93 | 0.883 |
| hsa-miR-200c-3p | 0.723 | 0.512 | 0.642 |
| hsa-miR-107 | 0.423 | 0.777 | 0.702 |
| hsa-miR-652-3p | 0.58 | 0.502 | 0.98 |
| hsa-miR-28-5p | 0.981 | 0.824 | 0.826 |
| hsa-miR-22-5p | 0.281 | 0.638 | 0.853 |
| hsa-miR-31-5p | 0.83 | 0.733 | 0.777 |
| hsa-miR-26b-5p | 0.653 | 0.771 | 0.894 |
| hsa-miR-192-5p | 0.542 | 0.726 | 0.839 |
| hsa-miR-151a-5p | 0.844 | 0.714 | 0.242 |
| hsa-miR-31-3p | 0.925 | 0.211 | 0.8 |

P values from robust regression controlling impact of factors as age, gender and grade on miRNA expression. P < 0.05 is considered as statistically significant
